# Clinical and Hormonal Characteristics and Short-Term Outcomes of Diazoxide Use in Hypoglycemic Neonates: A Retrospective Cohort Study

**DOI:** 10.3390/children13091157

**Published:** 2026-08-28

**Authors:** Sevil Bali, Burcu Cebeci

**Affiliations:** 1 Department of Pediatrics, Haseki Training and Research Hospital, University of Health Sciences, Istanbul 34265, Türkiye; 2 Department of Neonatology, Haseki Training and Research Hospital, University of Health Sciences, Istanbul 34265, Türkiye

**Keywords:** diazoxide, neonatal hypoglycemia, hyperinsulinism, insulin, C-peptide, pulmonary hypertension

## Abstract

**Highlights:**

**What are the main findings?**

- During concurrent hypoglycemia, diazoxide-treated infants had higher adjusted insulin (GMR 2.23) and C-peptide (GMR 1.59).
- Fluid retention requiring diuretic management occurred in 30.6% of treated infants; pulmonary hypertension was not documented on routine day 5–7 echocardiography in 36 treated infants.

**What are the implications of the main findings?**

- Critical-sample findings should be interpreted as evidence of inadequate insulin suppression in clinical context, not as proof that diazoxide exposure is a surrogate diagnosis of hyperinsulinism.
- Temporal anchoring of comorbidities is essential: much of the sepsis and respiratory-support burden in treated infants was already present before diazoxide initiation.

**Abstract:**

**Background/Objectives**: Diazoxide is used for persistent or recurrent hypoglycemia when the clinical and biochemical pattern is compatible with hyperinsulinism, but neonatal treatment populations are heterogeneous. We evaluated the clinical and hormonal characteristics and short-term outcomes associated with diazoxide use in hypoglycemic neonates. **Methods**: This single-center retrospective cohort included 124 hypoglycemic neonates treated between January 2020 and January 2026; 36 received diazoxide and 88 did not. Critical hormonal samples were obtained concurrently with glucose < 47 mg/dL; in treated infants, sampling preceded the first diazoxide dose. Hormonal effects were estimated with parsimonious multivariable models. Diazoxide-selection factors were examined with a reduced logistic model and Firth sensitivity analysis. **Results**: Treated infants were more frequently preterm (80.6% vs. 52.3%; *p* = 0.003) and had greater pre-treatment sepsis and ventilation burden. Severe hypoglycemia < 25 mg/dL did not differ significantly (27.8% vs. 17.0%; *p* = 0.176). Insulin was higher with diazoxide (median 6.05 vs. 2.77 mU/L; adjusted GMR 2.23, 95% CI 1.38–3.61; *p* = 0.001), as was C-peptide (adjusted GMR 1.59, 95% CI 1.03–2.47; *p* = 0.038). Among treated infants, 35/36 had insulin > 1.25 mU/L; 17/29 with available maximum glucose-infusion-rate data required >8 mg/kg/min. Diazoxide began at a median postnatal age of 10.5 days; 30/36 infants remained on therapy at discharge. Fluid retention requiring diuretics occurred in 11/36 (30.6%) versus 11/88 (12.5%; *p* = 0.017). Pulmonary hypertension was not documented on routine day 5–7 echocardiography (0/36; exact 95% CI 0.0–9.7%). **Conclusions**: Diazoxide use identified a clinically vulnerable, treatment-selected subgroup with evidence of inadequate insulin suppression during hypoglycemia. The data do not establish uniformly confirmed hyperinsulinism. Fluid retention was the main documented treatment-period adverse event; rare or later cardiopulmonary complications cannot be excluded by this retrospective cohort.

## 1. Introduction

Neonatal hypoglycemia is common in infants with prematurity, abnormal fetal growth, maternal diabetes, and perinatal illness. No single glucose concentration reliably separates physiological metabolic transition from pathological hypoglycemia; therefore, operational thresholds and the pattern of severity, recurrence, persistence, symptoms, and alternative-fuel availability are used to guide evaluation and treatment [1,2,3,4,5]. Severe or recurrent hypoglycemia is clinically important because the neonatal brain has high glucose requirements and may have limited ketone availability, particularly when insulin action is inappropriate [3,6,7,8].

Hyperinsulinism is a major cause of persistent or treatment-refractory hypoglycemia. Inappropriate insulin action during hypoglycemia suppresses hepatic glucose production, lipolysis, and ketogenesis. Diagnosis should therefore integrate the critical-sample glucose concentration with insulin or C-peptide, beta-hydroxybutyrate, free fatty acids, glucose infusion requirement, and the glycemic response to glucagon rather than depend on one biomarker alone [2,3,4,5,9,10]. Perinatal stress-associated hyperinsulinism is particularly relevant in neonatal intensive care because affected infants may be preterm, growth restricted, exposed to preeclampsia or asphyxia, or burdened by other morbidities [3,4,5]. Prolonged transitional hypoglycemia has also been characterized as a distinct clinical syndrome in which glycemic instability persists well beyond the expected transition period [11].

Diazoxide opens ATP-sensitive potassium channels in pancreatic beta cells and suppresses insulin secretion. International guidance recommends 5–15 mg/kg/day for established hyperinsulinism and emphasizes fluid and cardiopulmonary surveillance because sodium–water retention and pulmonary hypertension are clinically important concerns in neonates [4,12,13,14,15,16,17,18]. However, real-world NICU use may reflect clinician assessment of recurrent hypoglycemia, glucose requirements, available critical-sample findings, and comorbidity rather than a uniform diagnostic pathway.

We therefore evaluated a tertiary-NICU cohort to describe the clinical and hormonal characteristics associated with diazoxide use, quantify the completeness of retrospectively available biochemical evidence, temporally separate selected pre- and post-treatment morbidities, and describe short-term treatment-period outcomes without assuming that diazoxide exposure is a surrogate diagnosis of confirmed hyperinsulinism.

## 2. Materials and Methods

### 2.1. Study Design and Setting

This single-center retrospective observational cohort study was conducted in the neonatal intensive care unit (NICU) of University of Health Sciences, Haseki Training and Research Hospital, Istanbul, Türkiye. Medical records and electronic hospital data were reviewed for the period 1 January 2020 to 30 January 2026. The study is reported in accordance with the Strengthening the Reporting of Observational Studies in Epidemiology (STROBE) statement [19].

The study was conducted in accordance with the Declaration of Helsinki and approved by the Haseki Training and Research Hospital Non-Interventional Clinical Research Ethics Committee (Decision No. 101-2025; 25 June 2025). Written informed consent had been obtained from parents or legal guardians at hospital admission using the institutional inpatient consent form, which authorizes the use of anonymized clinical and medical-record data for scientific research and publication.

### 2.2. Cohort Selection and Exposure Definition

**Figure 1:**
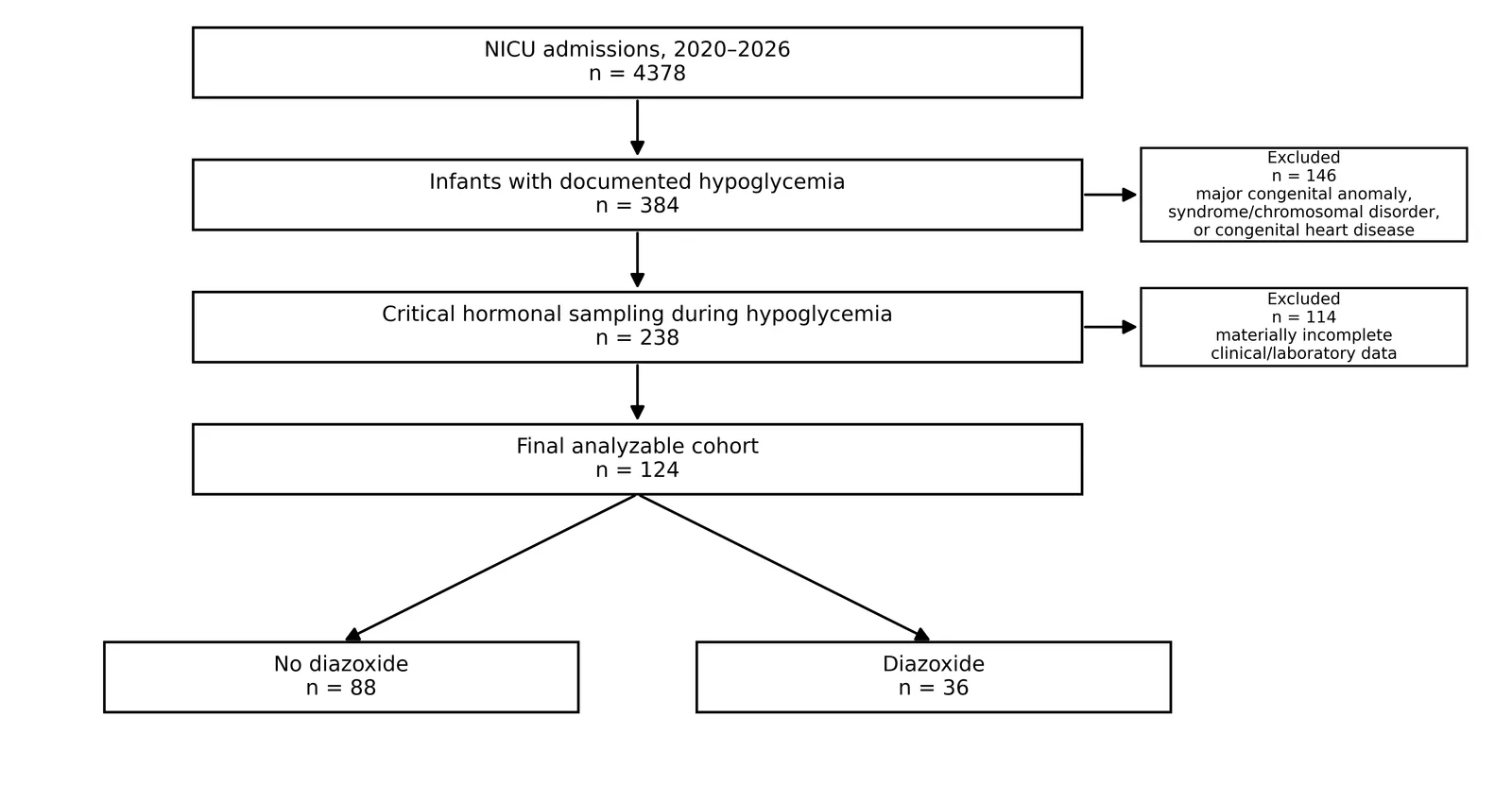
Study flow diagram.

During the study interval, 4378 neonates were admitted to the NICU and 384 had documented hypoglycemia. Among these, 146 were excluded because of major congenital anomaly, syndrome/chromosomal disorder, or congenital heart disease. Critical hormonal sampling during hypoglycemia was available in 238 infants; 114 were subsequently excluded because clinical or laboratory data were materially incomplete for the planned analyses. Patient-level source records were verified to ensure a single observation per infant. The final cohort therefore comprised 124 unique infants: 88 not exposed to diazoxide and 36 exposed to diazoxide (Figure 1).

A plasma or blood glucose concentration < 47 mg/dL was used as a uniform operational threshold to identify the study cohort; it was not intended to define pathological hypoglycemia independently of postnatal age or clinical context. Infants were classified according to whether diazoxide was administered during the neonatal admission. Treatment allocation was not prospectively protocolized and reflected treating clinicians’ assessment of persistence or recurrence, glucose severity, biochemical findings, glucose requirement, and comorbidities.

### 2.3. Critical-Sample Timing and Retrospective Hyperinsulinism Criteria

Glucose and hormonal measurements were obtained concurrently during the documented hypoglycemic episode, and all analyzed glucose values were <47 mg/dL. In diazoxide-treated infants, the critical sample was drawn immediately before the first diazoxide dose; therefore, the postnatal age of this qualifying hypoglycemic episode was the recorded diazoxide-initiation day. The exact postnatal age of the corresponding qualifying sample was not systematically retrievable for infants who were not treated with diazoxide. Feeding status and concurrent intravenous dextrose exposure at the exact sampling time could not be reconstructed reliably for every infant.

For a descriptive retrospective assessment aligned with international hyperinsulinism guidance [4], insulin > 1.25 mU/L and C-peptide > 0.5 ng/mL during hypoglycemia were treated as evidence of inadequate suppression of insulin secretion, while a maximum glucose infusion rate (GIR) > 8 mg/kg/min in neonates was treated as evidence supporting excessive insulin action. These criteria were not used to reclassify the cohort as confirmed hyperinsulinism because beta-hydroxybutyrate, free fatty acids, quantitative glucagon responses, and genetic testing were not systematically retrievable. Glucagon administration itself was recorded when available, but the post-glucagon glucose increment was not available.

### 2.4. Diazoxide Protocol and Cardiopulmonary Surveillance

Diazoxide was initiated at 5 mg/kg/day. If hypoglycemia persisted after treatment initiation, defined in local practice by recurrent glucose values < 60 mg/dL, the dose was increased according to glycemic response and tolerance, up to a maximum recorded dose of 15 mg/kg/day. The postnatal day of initiation, whether dose escalation occurred, the postnatal day of escalation, the maximum/final recorded dose, glucagon use, maximum GIR when retrievable, and whether diazoxide was continued at discharge were recorded. Total diazoxide treatment duration was not analyzed because retrospective documentation did not reliably distinguish inpatient from post-discharge treatment duration.

All 36 treated infants underwent routine echocardiography by pediatric cardiology 5–7 days after diazoxide initiation. Pulmonary hypertension was determined from the cardiologist’s integrated echocardiographic assessment. Repeat echocardiography beyond the routine day 5–7 study was not systematically captured. The median available in-hospital observation period after diazoxide initiation was derived from the recorded admission duration and initiation day.

### 2.5. Clinical Variables and Operational Outcome Definitions

Demographic and perinatal variables included gestational age, birth weight, sex, preterm birth, small-for-gestational-age (SGA) and large-for-gestational-age status, maternal diabetes, preeclampsia, and antenatal corticosteroid exposure. Laboratory variables included glucose, insulin, C-peptide, cortisol, ACTH, growth hormone, TSH, free thyroxine, blood counts, aminotransferases, and creatinine. Severe hypoglycemia was defined as glucose < 25 mg/dL.

Sepsis was defined as a documented neonatal clinical diagnosis encompassing both culture-proven sepsis and culture-negative clinical sepsis. In diazoxide-treated infants, sepsis and mechanical ventilation were separately coded as present before and after the first diazoxide dose; PDA was coded according to documented status before treatment. In the untreated comparison group, the corresponding routinely recorded admission variables were used because no treatment index time existed. PDA was defined by the documented clinical/echocardiographic diagnosis. Necrotizing enterocolitis (NEC) was defined as a documented Bell stage II or higher event.

Fluid retention was defined as documented edema or volume retention requiring active diuretic management during the relevant admission/treatment period. Among treated infants, recorded diuretic treatment for fluid retention consisted of furosemide or spironolactone; routine prophylactic diuretic use was not inferred from the dataset. Laboratory abnormalities were not consistently time-indexed to diazoxide initiation and were therefore not analyzed as drug-attributable toxicity; instead, treatment discontinuation because of a documented adverse event was recorded as a pragmatic treatment-limiting outcome.

Hormonal measurements obtained at a glucose concentration below 47 mg/dL included insulin, C-peptide, cortisol, adrenocorticotropic hormone (ACTH), growth hormone, thyroid-stimulating hormone (TSH), and free thyroxine. Insulin, C-peptide, cortisol, TSH, and free thyroxine were analyzed on an Atellica platform; ACTH and growth hormone were measured using an IMMULITE 2000 analyzer (Siemens Healthineers, Erlangen, Germany). Hormone-specific denominators were reported because critical-sample completeness varied.

### 2.6. Study Size and Birth-Weight Power Calculation

No a priori sample-size calculation determined enrollment because this was a retrospective cohort and all eligible infants with analyzable records were included. For the birth-weight comparison, the between-group mean difference is reported with its 95% confidence interval to indicate the magnitude and precision of the estimated difference.

### 2.7. Statistical Analysis

Continuous variables are reported as mean ± standard deviation when approximately symmetric and as median (interquartile range) otherwise; categorical variables are reported as n (%). Between-group comparisons used Welch’s *t* test for approximately symmetric continuous variables, the Mann–Whitney U test for skewed continuous variables, and chi-square or Fisher’s exact tests for categorical variables as appropriate.

Hormonal analyses used available cases. Insulin, C-peptide, cortisol, growth hormone, and TSH were log transformed and analyzed with multivariable linear regression using HC3 heteroscedasticity-robust standard errors. Adjusted effects are presented as geometric mean ratios (GMRs). Free thyroxine was modeled on the original scale and is reported as an adjusted mean difference. To avoid structurally overlapping glucose-derived covariates, hypoglycemia deficit was removed from all adjusted models. The prespecified parsimonious hormonal adjustment set comprised gestational age, sex, SGA, maternal diabetes, preeclampsia, antenatal steroid exposure, and severe hypoglycemia. ACTH was not adjusted because only 30 observations were available.

Variables associated with diazoxide selection were evaluated using a reduced clinically selected logistic model containing gestational age, SGA, severe hypoglycemia, pre-treatment PDA, and pre-treatment sepsis. Because only 36 infants received diazoxide, standard logistic estimates were accompanied by Firth penalized-likelihood sensitivity estimates; the model is descriptive and not a validated prediction tool.

A propensity-score sensitivity analysis was rebuilt using only variables available before treatment selection: gestational age, birth weight, sex, SGA, maternal diabetes, preeclampsia, antenatal steroids, and severe hypoglycemia. Stabilized inverse-probability-of-treatment weights (IPTW) were trimmed at the 1st and 99th percentiles. Propensity-score range, weight distribution, effective sample size (ESS), and standardized mean differences (SMDs) are reported in Supplementary Table S1 and Figure S1. Because residual imbalance remained for gestational age and sex after trimmed IPTW and because an equivalent post-treatment index time could not be defined in untreated infants, weighted post-treatment outcome comparisons were not interpreted as causal estimates. Two-sided *p* < 0.05 was considered statistically significant; secondary analyses were interpreted as exploratory without multiplicity adjustment.

## 3. Results

### 3.1. Study Population and Clinical Characteristics

The final cohort comprised 124 hypoglycemic neonates: 36 (29.0%) received diazoxide and 88 (71.0%) did not. Treated infants were more frequently preterm (80.6% vs. 52.3%; *p* = 0.003), although mean gestational age and birth weight did not reach statistical significance. Severe hypoglycemia < 25 mg/dL was not significantly different between groups (27.8% vs. 17.0%; *p* = 0.176). Pre-treatment PDA, sepsis, and mechanical ventilation were more frequent in treated infants; hemoglobin and hematocrit were lower, while maternal diabetes, preeclampsia, and antenatal steroid exposure were more common (Table 1).

The mean birth weight was 2345 ± 954 g in untreated infants and 2070 ± 915 g in diazoxide-treated infants. The mean difference (diazoxide minus no diazoxide) was −275 g (95% CI −640 to 91 g; *p* = 0.139).

### 3.2. Critical-Sample Hormonal Findings and Diagnostic Completeness

**Figure 2:**
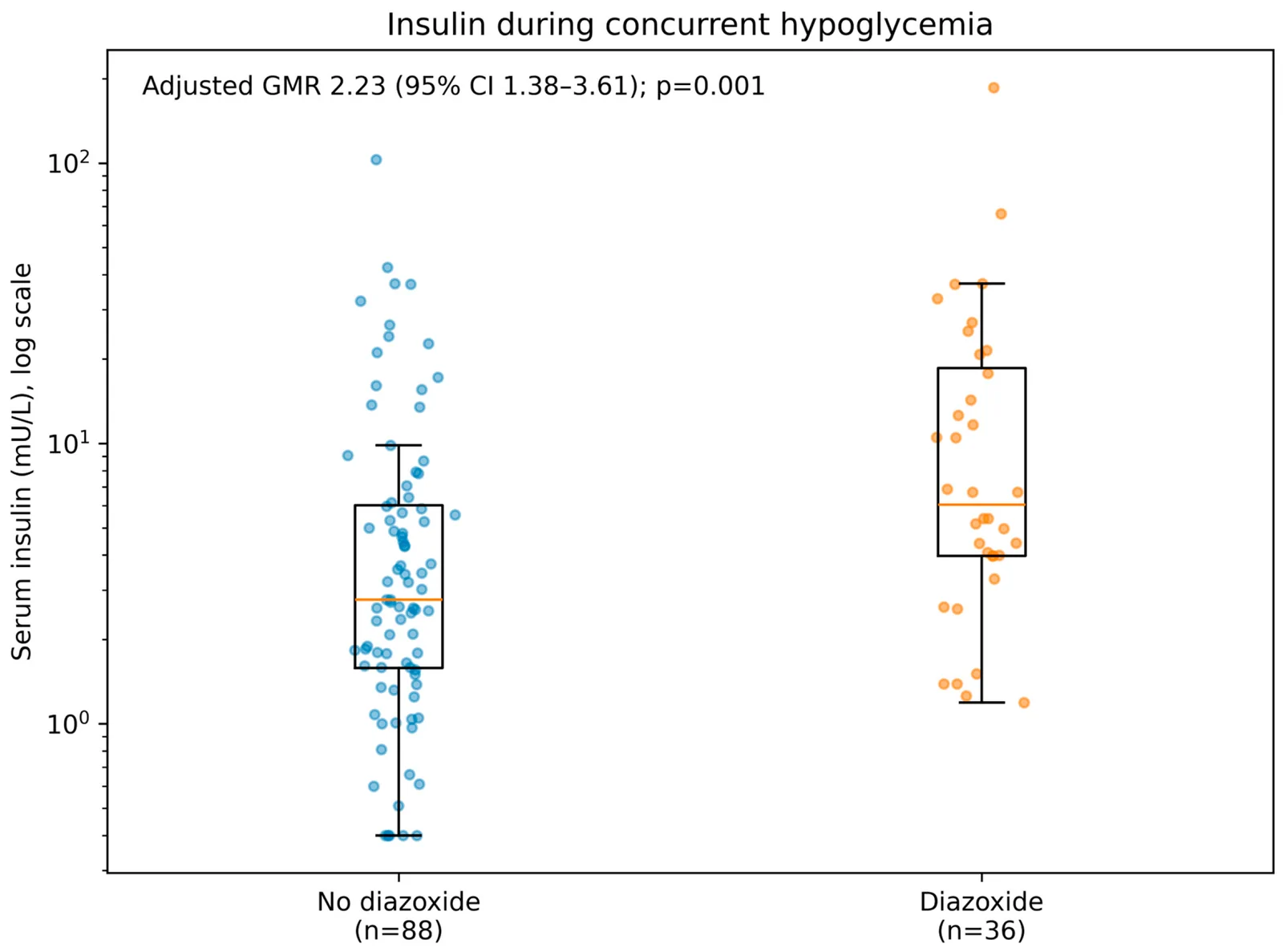
Serum insulin concentrations during concurrent hypoglycemia according to diazoxide exposure. The y-axis is logarithmic. The adjusted GMR is from log-linear regression with HC3 robust standard errors adjusted for gestational age, sex, SGA, maternal diabetes, preeclampsia, antenatal steroid exposure, and severe hypoglycemia.

All 124 infants had simultaneous glucose and insulin measurements during glucose < 47 mg/dL. Median insulin was 6.05 mU/L in treated infants versus 2.77 mU/L in untreated infants (*p* < 0.001). Insulin concentrations during concurrent hypoglycemia are given in Figure 2. After parsimonious adjustment, the geometric mean insulin concentration was 2.23-fold higher with diazoxide exposure (95% CI 1.38–3.61; *p* = 0.001). C-peptide was also higher after adjustment (GMR 1.59, 95% CI 1.03–2.47; *p* = 0.038). Cortisol, growth hormone, TSH, and free thyroxine did not differ after adjustment. ACTH was lower in a sparse crude comparison (30 available observations; *p* = 0.041) but was not adjusted and is interpreted as exploratory (Table 2).

Among treated infants, 35/36 (97.2%) had insulin > 1.25 mU/L during hypoglycemia. C-peptide was >0.5 ng/mL in 30/33 (90.9%) with available measurements; the one treated infant with insulin ≤ 1.25 mU/L had C-peptide 1.52 ng/mL, so all 36 treated infants met at least one available secretion criterion. Maximum GIR was retrievable in 29/36 (80.6%), with a median of 10 mg/kg/min (IQR 8–12); 17/29 (58.6%) exceeded 8 mg/kg/min. Glucagon had been administered in 17/36 (47.2%), but a quantitative glycemic response could not be reconstructed. In the untreated group, insulin > 1.25 mU/L was also common (71/88, 80.7%), underscoring that the comparator should not be interpreted as a uniformly non-hyperinsulinemic control group.

### 3.3. Diazoxide Treatment and Short-Term Safety

Diazoxide was started at 5 mg/kg/day at a median postnatal age of 10.5 days (IQR 4.0–20.2). Dose escalation was required in 22/36 infants (61.1%) when glucose remained <60 mg/dL; among those escalated, the median postnatal day of escalation was 15.5 (IQR 11.0–24.8). The median dose at discharge was 7.5 mg/kg/day (IQR 5.0–10.0; range 5–15), and 30/36 infants (83.3%) remained on diazoxide at discharge (Table 3).

Fluid retention/edema requiring diuretic management was documented in 11/36 treated infants (30.6%) compared with 11/88 untreated infants (12.5%; *p* = 0.017; crude OR 3.08, 95% CI 1.19–7.96). Management in treated infants consisted of furosemide in nine and spironolactone in two. All treated infants underwent routine echocardiography on treatment day 5–7; no pulmonary hypertension was documented (0/36; exact 95% CI 0.0–9.7%). Baseline echocardiography had been performed in 12/36. The derived median available in-hospital surveillance after diazoxide initiation was 18.0 days (IQR 9.8–34.5). No NEC or adverse event requiring diazoxide discontinuation was documented during the available treatment-period surveillance.

### 3.4. Temporal Anchoring of Morbidity

Temporally anchored data demonstrated that much of the morbidity burden in treated infants preceded diazoxide initiation. Sepsis was already present in 25/36 (69.4%) before the first dose. Among the 11 infants without pre-treatment sepsis, one developed a subsequently documented sepsis episode (9.1%; exact 95% CI 0.2–41.3%). Mechanical ventilation was already required in 27/36 (75.0%) before treatment; among the nine infants not ventilated before diazoxide, none subsequently required ventilation (0/9; exact 95% CI 0.0–33.6%). These data support descriptive temporal separation rather than attributing the high crude morbidity prevalence to diazoxide exposure (Table 4).

### 3.5. Reduced Model of Diazoxide Selection

**Figure 3:**
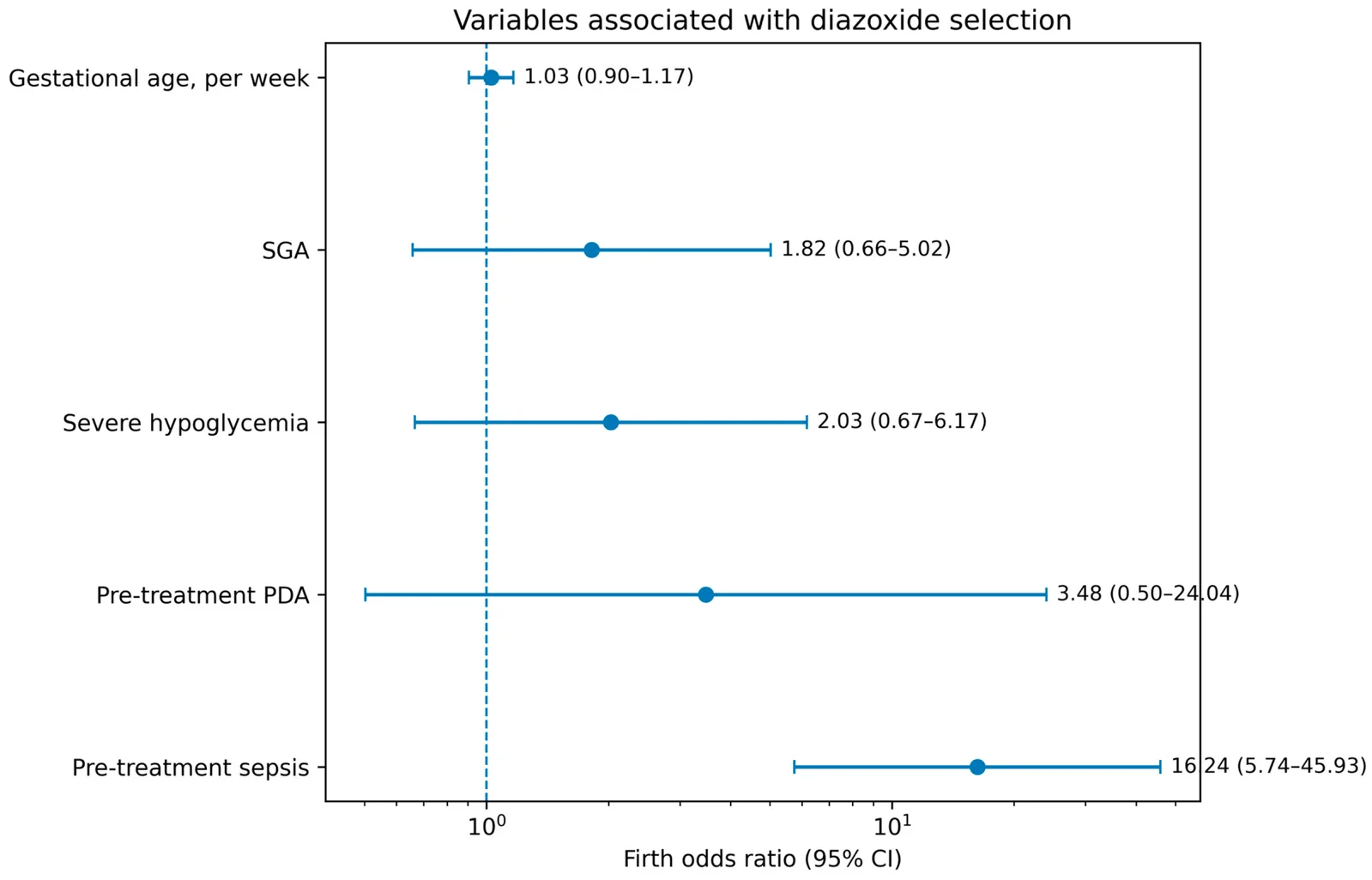
Firth penalized-logistic estimates for the reduced clinically selected model of diazoxide selection. Error bars represent approximate 95% confidence intervals. The model is descriptive and not a validated prediction tool.

A five-variable clinically selected model was used to limit overfitting in the treatment-selection analysis. In both standard and Firth analyses, pre-treatment sepsis was strongly associated with diazoxide selection, whereas gestational age, SGA, severe hypoglycemia, and pre-treatment PDA had wide confidence intervals crossing unity (Table 5; Figure 3). These estimates describe the clinical selection context and should not be interpreted as causal determinants or a validated prediction model.

### 3.6. Propensity-Score Sensitivity Diagnostics

The propensity-score sensitivity analysis used only pre-treatment/perinatal variables. Propensity scores ranged from 0.052 to 0.896. Stabilized untrimmed weights ranged from 0.324 to 3.131; after 1st–99th percentile trimming, weights ranged from 0.366 to 2.450. The effective sample size was 106.3 overall (28.8 treated and 77.4 untreated). Most covariates achieved an absolute SMD < 0.10, but residual imbalance remained for gestational age (SMD 0.130) and male sex (SMD 0.220). Because this balance criterion was not fully met and because untreated infants lacked a comparable post-treatment index time, weighted post-treatment clinical outcome estimates were not retained as causal results. Detailed diagnostics are provided in Supplementary Table S1 and Figure S1.

## 4. Discussion

This study describes the clinical and hormonal phenotype of hypoglycemic neonates selected for diazoxide treatment in a tertiary intensive-care setting. Hypoglycemic neonates selected for diazoxide had higher insulin during concurrent hypoglycemia, and C-peptide was also higher after adjustment. Severe hypoglycemia was not significantly different between groups, and temporally anchored data showed that much of the sepsis and respiratory-support burden in treated infants was already present before diazoxide initiation. These findings support a treatment-selection phenotype rather than a uniformly confirmed hyperinsulinism cohort.

The insulin finding should be interpreted as inadequate suppression of insulin secretion rather than simply as a higher concentration or as evidence of greater insulin action. International guidance emphasizes that insulin and C-peptide are less definitive than evidence of insulin action such as suppressed beta-hydroxybutyrate/free fatty acids, an increased GIR, or a large glycemic response to glucagon [4]. In our treated group, 35/36 infants had insulin > 1.25 mU/L and all 36 had at least one available secretion marker above the guideline threshold. However, insulin > 1.25 mU/L was also present in 80.7% of untreated infants, making it inappropriate to describe the untreated group as confirmed non-hyperinsulinemic controls. The comparison is therefore between treatment-selected and non-treated hypoglycemic neonates, not between confirmed HI and confirmed non-HI.

The additional diagnostic-completeness analysis reinforces this distinction. Maximum GIR was retrievable in 29/36 treated infants and exceeded 8 mg/kg/min in 58.6%. Glucagon had been used in 47.2%, but the quantitative glucose increment could not be recovered. BOHB, free fatty acids, and genetic testing were not sufficiently standardized for patient-level analysis. These gaps prevent retrospective confirmation of the full international diagnostic framework, even though the available biochemical pattern in the treated group frequently supported inadequate insulin suppression [4,9,10,20].

C-peptide was also higher in the diazoxide-treated group in our study. Ferrara et al. demonstrated that C-peptide can be diagnostically useful, including when insulin is low or undetectable, but also showed that no single biomarker is perfectly sensitive [9]. More recently, Petkovic et al. reported high diagnostic performance for a C-peptide threshold in a pediatric congenital-hyperinsulinism cohort; that estimate was not neonatal-specific and should not be transferred directly to critically ill newborns [10]. In transient neonatal hyperinsulinism, Davidov et al. found that higher C-peptide and greater glucose requirements identified infants more likely to receive diazoxide [20]. The absence of between-group differences in cortisol and growth hormone should not be interpreted as excluding endocrine deficiency because measurements during hypoglycemia have limited diagnostic specificity [21]. These findings reinforce the need to interpret each hormone as one component of a complete critical sample.

Diazoxide was initiated later than in trials of early low-dose treatment for transitional or recurrent neonatal hypoglycemia. The median initiation age was 10.5 days, which is consistent with escalation after persistence or recurrence rather than universal treatment of early transitional low glucose. The starting dose was 5 mg/kg/day, 61.1% required escalation, and the final recorded dose remained within the 5–15 mg/kg/day guideline range [4,12]. In the NeoGluCO randomized trial of 74 late-preterm and term newborns, low-dose diazoxide did not significantly shorten the prespecified time to complete resolution of hypoglycemia, but it reduced hypoglycemic episodes and duration, blood-glucose testing, time to enteral bolus feeding, and time to withdrawal of intravenous fluids [22]. An earlier randomized trial in low-birth-weight neonates with hyperinsulinemic hypoglycemia similarly reported earlier glycemic control and shorter duration of intravenous dextrose without apparent adverse effects [23]. Notably, our median discharge dose of 7.5 mg/kg/day sits at the lower end of the guideline range, in keeping with accumulating evidence that low-dose regimens are effective in transient hyperinsulinism while limiting fluid-related toxicity [24]. Because a reliable total treatment-duration variable was not available and standardized post-treatment glycemic episode counts were not captured, the present study should not be interpreted as a comparative efficacy study.

Fluid retention occurred in 30.6% of treated infants, compared with 12.5% of untreated infants managed in the same unit. A 2021 meta-analysis reported pooled rates of approximately 20% for fluid retention and lower frequencies for edema and pulmonary hypertension, although definitions and populations varied substantially [25]. The 2026 multicenter cohort by Collins et al., including 545,065 infants from 345 neonatal intensive care units, found diazoxide exposure in 0.16% of infants; after exposure, 13% began a new diuretic course, 10% required new oxygen supplementation, and 3% required new ventilatory support [15]. Differences from our fluid-retention rate may relate to our high prevalence of prematurity and comorbidity, local management practices, and the distinction between a documented adverse event and initiation of supportive therapy. The comparator rate of 12.5% in our own untreated infants suggests that a substantial proportion of edema in this population is attributable to underlying illness rather than to diazoxide. Regardless of the exact attributable fraction, daily weight and fluid-balance review, respiratory assessment, and judicious thiazide use remain central to neonatal diazoxide care [4,12].

Pulmonary hypertension was not documented in any of the 36 treated infants on the protocolized day 5–7 echocardiogram. The exact 95% confidence interval for a zero-event result extends to 9.7%, so this finding cannot exclude a clinically important rare risk. Baseline echocardiography was available in only one third of treated infants, and repeat imaging beyond the routine early study was not systematically captured. Observational series, pharmacovigilance data, and neonatal reports identify prematurity, perinatal stress, underlying cardiac disease, and fluid overload as important contexts for diazoxide-associated pulmonary hypertension [13,14,15,16,17,18]. The influence of surveillance intensity is illustrated by two contrasting datasets. Duggal et al. reported pulmonary hypertension in 12 of 36 infants (33%) who had echocardiography after diazoxide initiation, a much higher figure than clinically ascertained series [18]. Conversely, in the NeoGluCO trial, among newborns who underwent protocolized cardiac ultrasonography (29 diazoxide, 33 placebo), none had pulmonary hypertension or cardiac impairment by predefined criteria [22]. Our null result resembles the trial experience, but our population was substantially more preterm and comorbid, and echocardiography was generally performed about one week after initiation; later-onset disease could have been missed if symptoms or repeat imaging were not recorded. Baseline and post-treatment echocardiography, together with active fluid surveillance, remains appropriate in high-risk infants.

Necrotizing enterocolitis has also been reported in diazoxide-exposed neonates, although causality remains uncertain and underlying illness may contribute [26,27]. A systematic appraisal of this question concluded that the available evidence is limited and confounded, and that a causal relationship has not been established [28]. No NEC occurred among our treated infants.

The temporal reconstruction of sepsis and ventilation is particularly important. More than two thirds of treated infants already had sepsis and three quarters were already receiving mechanical ventilation before diazoxide. Among infants free of these conditions before treatment, only one out of eleven developed a later sepsis episode and none of nine required new ventilation. These small denominators preclude strong safety inference, but they show why crude admission-level morbidity cannot be attributed to diazoxide without careful time ordering.

The statistical strategy was intentionally conservative. With only 36 treated infants, a high-dimensional logistic model would be vulnerable to overfitting, particularly if overlapping glucose-derived terms were included. The reduced five-variable model and Firth sensitivity analysis address this concern, although the very large pre-treatment sepsis association should still be viewed as a marker of the clinical selection context rather than a treatment indication in itself. Similarly, the propensity-score analysis is reported as a diagnostic sensitivity analysis rather than as a causal outcome model. Baseline-only weighting improved balance substantially, but gestational age and sex remained above the prespecified SMD threshold and untreated infants lacked a symmetric index time for post-treatment outcomes [29].

Several limitations remain. This was a retrospective, single-center study based on non-protocolized treatment decisions, limiting generalizability to centers with different hypoglycemia evaluation, diazoxide thresholds, critical-sample practices, and cardiopulmonary surveillance. Although glucose and hormones were sampled concurrently and before diazoxide in treated infants, feeding status and exact intravenous dextrose exposure at the critical sample could not be reconstructed for every infant. The exact postnatal age of the qualifying sample was not systematically retrievable in untreated infants. BOHB, free fatty acids, quantitative glucagon response, and genetic data were incomplete or not standardized. Hormone analyses used available cases, and ACTH was particularly sparse. Baseline echocardiography was incomplete, and a single early post-treatment echocardiogram cannot exclude later pulmonary hypertension. The treated sample remained modest, confidence intervals for rare events were wide, and no long-term neurodevelopmental outcomes were available.

Strengths include inclusion of all eligible infants across six years, patient-level source-record verification ensuring a single observation per infant, simultaneous glucose-hormone sampling, explicit reporting of diagnostic completeness, removal of structurally collinear glucose variables, reduction of the treatment-selection model with Firth sensitivity analysis, temporal separation of key morbidities relative to diazoxide initiation, complete day 5–7 echocardiographic surveillance in treated infants, and transparent propensity-score diagnostics rather than causal overinterpretation. The cohort also reflects a clinically relevant neonatal intensive care population that is more premature and medically fragile than many trial populations.

## 5. Conclusions

Diazoxide-treated hypoglycemic neonates were a treatment-selected, clinically vulnerable subgroup with higher insulin and C-peptide during concurrent hypoglycemia. Fluid retention requiring diuretic management was the main documented treatment-period adverse event. Pulmonary hypertension was not detected on routine day 5–7 echocardiography in 36 treated infants, but rare or later events cannot be excluded. Temporally anchored analyses show that substantial sepsis and respiratory morbidity preceded treatment. Multicenter prospective studies using complete critical samples and standardized longitudinal cardiopulmonary surveillance are needed.

## Figures and Tables

**Table 1:** Demographic, clinical, laboratory, and maternal characteristics in the final patient-level cohort.

Variable	All Infants (N = 124)	No Diazoxide (N = 88)	Diazoxide (N = 36)	*p* Value
Gestational age, weeks	34.8 ± 4.0	35.1 ± 4.3	33.8 ± 3.2	0.070
Preterm birth, n (%)	75 (60.5)	46 (52.3)	29 (80.6)	0.003
Birth weight, g	2265 ± 948	2345 ± 954	2070 ± 915	0.139
Male sex, n (%)	84 (67.7)	55 (62.5)	29 (80.6)	0.051
SGA, n (%)	47 (37.9)	33 (37.5)	14 (38.9)	0.885
LGA, n (%)	4 (3.2)	3 (3.4)	1 (2.8)	1.000
PDA, n (%) †	8 (6.5)	3 (3.4)	5 (13.9)	0.045
Sepsis, n (%) †	36 (29.0)	11 (12.5)	25 (69.4)	<0.001
Mechanical ventilation, n (%) †	62 (50.0)	35 (39.8)	27 (75.0)	<0.001
Glucose at critical sample, mg/dL	34.0 (26.8–39.0)	34.0 (27.0–38.2)	34.5 (23.5–40.0)	0.873
Severe hypoglycemia (<25 mg/dL), n (%)	25 (20.2)	15 (17.0)	10 (27.8)	0.176
Creatinine, mg/dL	0.66 (0.51–0.78)	0.66 (0.55–0.78)	0.62 (0.33–0.79)	0.194
ALT, U/L	11 (8–16)	11 (7–15)	12 (9–17)	0.052
AST, U/L	50 (32–66)	51 (31–64)	46 (32–66)	0.788
Leukocyte count, ×10^3^/µL	14.5 ± 7.0	14.9 ± 7.0	13.7 ± 7.0	0.386
Platelet count, ×10^3^/µL	217 ± 128	206 ± 108	243 ± 167	0.225
Hemoglobin, g/dL	17.2 ± 4.1	18.1 ± 4.0	15.2 ± 3.9	<0.001
Hematocrit, %	51.4 ± 12.2	53.6 ± 11.6	46.2 ± 12.1	0.003
Maternal diabetes, n (%)	18 (14.5)	8 (9.1)	10 (27.8)	0.007
Antenatal steroids, n (%)	24 (19.4)	11 (12.5)	13 (36.1)	0.003
Preeclampsia, n (%)	21 (16.9)	10 (11.4)	11 (30.6)	0.010

Values are mean ± standard deviation, median (interquartile range), or n (%). † For diazoxide-treated infants, PDA, sepsis, and mechanical ventilation represent status documented before the first diazoxide dose; in untreated infants, the corresponding routinely recorded admission variable is shown because no treatment index time exists. SGA, small for gestational age; LGA, large for gestational age; PDA, patent ductus arteriosus.

**Table 2:** Hormonal measurements during concurrent hypoglycemia and adjusted effects.

Hormone	No Diazoxide, n/N; Median (IQR)	Diazoxide, n/N; Median (IQR)	Unadjusted *p*	Adjusted Effect (95% CI)	Adjusted *p*
Insulin, mU/L	88/88; 2.77 (1.58–6.02)	36/36; 6.05 (3.97–18.55)	<0.001	GMR 2.23 (1.38–3.61)	0.001
C-peptide, ng/mL	78/88; 1.49 (1.08–2.51)	33/36; 1.90 (1.52–4.60)	0.009	GMR 1.59 (1.03–2.47)	0.038
Cortisol, µg/dL	79/88; 9.66 (4.42–22.55)	35/36; 8.63 (5.33–16.85)	0.557	GMR 0.80 (0.47–1.33)	0.386
ACTH, pg/mL	20/88; 37.60 (16.60–58.50)	10/36; 14.50 (9.95–14.70)	0.041	Not modeled	-
Growth hormone, ng/mL	63/88; 16.90 (9.51–27.60)	22/36; 18.00 (11.68–26.20)	0.684	GMR 1.23 (0.69–2.19)	0.483
TSH, mIU/L	82/88; 7.20 (3.82–11.91)	36/36; 6.45 (4.37–12.74)	0.895	GMR 1.11 (0.72–1.70)	0.649
Free thyroxine, pmol/L	79/88; 16.90 (13.35–20.15)	34/36; 15.30 (12.43–18.27)	0.247	Mean difference 0.34 (−2.21 to 2.89)	0.792

Available-case denominators are shown. Adjusted models included gestational age, sex, SGA, maternal diabetes, preeclampsia, antenatal steroids, and severe hypoglycemia; hypoglycemia deficit was deliberately excluded. Insulin, C-peptide, cortisol, growth hormone, and TSH were log transformed and are reported as GMRs. ACTH was not adjusted because only 30 observations were available. CI, confidence interval; GMR, geometric mean ratio; IQR, interquartile range.

**Table 3:** Retrospective diagnostic completeness, diazoxide course, and treatment-period surveillance among treated infants.

Variable	Diazoxide Group (N = 36)	Interpretation
Critical glucose + insulin available	36/36 (100%)	All sampled concurrently at glucose < 47 mg/dL before first diazoxide dose
Insulin > 1.25 mU/L	35/36 (97.2%)	Evidence of inadequate insulin suppression
C-peptide available	33/36 (91.7%)	Available-case assessment
C-peptide > 0.5 ng/mL	30/33 (90.9%)	Evidence of inadequate insulin suppression
At least one insulin/C-peptide secretion criterion	36/36 (100%)	Does not by itself confirm HI
Maximum GIR available	29/36 (80.6%)	Median 10 (8–12) mg/kg/min
Maximum GIR > 8 mg/kg/min	17/29 (58.6%)	Supports excessive insulin action
Glucagon administered	17/36 (47.2%)	Quantitative glucose increment unavailable
Starting diazoxide dose	5 mg/kg/day	Local starting dose
Postnatal age at initiation, days	10.5 (4.0–20.2)	Median (IQR)
Dose escalation	22/36 (61.1%)	Used when glucose remained <60 mg/dL
Postnatal age at escalation, days	15.5 (11.0–24.8)	Among 22 escalated infants
Maximum/final recorded dose, mg/kg/day	7.5 (5.0–10.0); range 5–15	Median (IQR)
Diazoxide continued at discharge	30/36 (83.3%)	Treatment ongoing at discharge
Baseline echocardiography	12/36 (33.3%)	Before diazoxide
Routine post-initiation echocardiography	36/36 (100%)	Performed day 5–7
Pulmonary hypertension	0/36 (0%; 95% CI 0.0–9.7%)	Not documented during available surveillance
Fluid retention/edema requiring diuretic treatment	11/36 (30.6%)	Furosemide n = 9; spironolactone n = 2
NEC	0/36 (0%; 95% CI 0.0–9.7%)	No documented Bell stage II or higher event
Adverse event requiring diazoxide discontinuation	0/36 (0%; 95% CI 0.0–9.7%)	No treatment-limiting event documented

Values are n/N (%) or median (IQR). BOHB, free fatty acids, quantitative glucagon response, and genetic testing were not systematically retrievable and therefore are not presented as complete diagnostic criteria. GIR, glucose infusion rate; HI, hyperinsulinism; NEC, necrotizing enterocolitis.

**Table 4:** Timing of selected clinical events in diazoxide-treated infants.

Event	n/N (%)	Comment
PDA present before diazoxide	5/36 (13.9%)	Baseline/pre-treatment condition
Sepsis present before diazoxide	25/36 (69.4%)	Clinical diagnosis; culture-positive or culture-negative
Sepsis documented after diazoxide, any	10/36 (27.8%)	May include persistence/recurrent episodes in infants with pre-treatment sepsis
Incident post-treatment sepsis among those without pre-treatment sepsis	1/11 (9.1%)	Exact 95% CI 0.2–41.3%
Mechanical ventilation before diazoxide	27/36 (75.0%)	Baseline/pre-treatment respiratory support
Mechanical ventilation documented after diazoxide, any	17/36 (47.2%)	Includes continuing support from before treatment
Incident post-treatment ventilation among those not ventilated pre-treatment	0/9 (0%)	Exact 95% CI 0.0–33.6%
Fluid retention/edema during diazoxide exposure	11/36 (30.6%)	All managed with furosemide or spironolactone

The table separates conditions documented before treatment from events documented after treatment in the diazoxide group. It does not provide a causal comparison with untreated infants because no equivalent treatment index time exists in the comparator group.

**Table 5:** Reduced clinically selected logistic model of diazoxide selection.

Variable	Standard OR (95% CI)	*p* Value	Firth OR (95% CI)	*p* Value
Gestational age, per week	1.03 (0.91–1.17)	0.645	1.03 (0.90–1.17)	0.681
SGA	1.95 (0.68–5.60)	0.215	1.82 (0.66–5.02)	0.250
Severe hypoglycemia (<25 mg/dL)	2.04 (0.65–6.41)	0.222	2.03 (0.67–6.17)	0.214
Pre-treatment PDA	3.97 (0.54–29.28)	0.176	3.48 (0.50–24.04)	0.207
Pre-treatment sepsis	20.01 (6.80–58.90)	<0.001	16.24 (5.74–45.93)	<0.001

All variables shown were entered simultaneously. Firth estimates are provided as a small-sample/separation sensitivity analysis. The reduced model was selected to limit overfitting; hypoglycemia deficit and post-treatment variables were not included. CI, confidence interval; OR, odds ratio; PDA, patent ductus arteriosus; SGA, small for gestational age.

## Data Availability

The data presented in this study are available on reasonable request from the corresponding author. The data are not publicly available because of patient confidentiality and institutional restrictions.

## Supplementary Materials

The following supporting information can be downloaded at: https://www.mdpi.com/article/10.3390/children13091157/s1, Supplementary Table S1. Propensity-score sensitivity diagnostics; Supplementary Figure S1. Propensity-score covariate balance before and after 1st–99th percentile trimmed stabilized IPTW. The dashed vertical line indicates an absolute SMD of 0.10.

## Abbreviations

The following abbreviations are used in this manuscript: ACTH, adrenocorticotropic hormone; BOHB, beta-hydroxybutyrate; CI, confidence interval; ESS, effective sample size; FFA, free fatty acid; GIR, glucose infusion rate; GMR, geometric mean ratio; HI, hyperinsulinism; IPTW, inverse probability of treatment weighting; IQR, interquartile range; NEC, necrotizing enterocolitis; NICU, neonatal intensive care unit; OR, odds ratio; PDA, patent ductus arteriosus; SGA, small for gestational age; SMD, standardized mean difference; TSH, thyroid-stimulating hormone.

## References

[1] Adamkin D.H. (2011). Postnatal glucose homeostasis in late-preterm and term infants. Pediatrics.

[2] Thornton P.S., Stanley C.A., De Leon D.D., Harris D., Haymond M.W., Hussain K., Levitsky L.L., Murad M.H., Rozance P.J., Simmons R.A. (2015). Recommendations from the Pediatric Endocrine Society for evaluation and management of persistent hypoglycemia in neonates, infants, and children. J. Pediatr..

[3] Stanley C.A., Rozance P.J., Thornton P.S., De Leon D.D., Harris D., Haymond M.W., Hussain K., Levitsky L.L., Murad M.H., Simmons R.A. (2015). Re-evaluating transitional neonatal hypoglycemia: Mechanism and implications for management. J. Pediatr..

[4] De Leon D.D., Arnoux J.B., Banerjee I., Bergada I., Bhatti T., Conwell L.S., Fu J., Flanagan S.E., Gillis D., Meissner T. (2024). International guidelines for the diagnosis and management of hyperinsulinism. Horm. Res. Paediatr..

[5] Lord K., De Leon D.D. (2024). Approach to the neonate with hypoglycemia. J. Clin. Endocrinol. Metab..

[6] McKinlay C.J.D., Alsweiler J.M., Ansell J.M., Anstice N.S., Chase J.G., Gamble G.D., Harris D.L., Jacobs R.J., Jiang Y., Paudel N. (2015). Neonatal glycemia and neurodevelopmental outcomes at 2 years. N. Engl. J. Med..

[7] McKinlay C.J.D., Alsweiler J.M., Anstice N.S., Burakevych N., Chakraborty A., Chase J.G., Gamble G.D., Harris D.L., Jacobs R.J., Jiang Y. (2017). Association of neonatal glycemia with neurodevelopmental outcomes at 4.5 years. JAMA Pediatr..

[8] Shah R., Harding J., Brown J., McKinlay C. (2019). Neonatal glycaemia and neurodevelopmental outcomes: A systematic review and meta-analysis. Neonatology.

[9] Ferrara C., Patel P., Becker S., Stanley C.A., Kelly A. (2016). Biomarkers of insulin for the diagnosis of hyperinsulinemic hypoglycemia in infants and children. J. Pediatr..

[10] Petkovic G., Park J., Collingwood C., Senniappan S., Didi M. (2025). Biomarkers and diagnostic thresholds for congenital hyperinsulinism. Clin. Endocrinol..

[11] Bailey M.J., Rout A., Harding J.E., Alsweiler J.M., Cutfield W.S., McKinlay C.J.D. (2021). Prolonged transitional neonatal hypoglycaemia: Characterisation of a clinical syndrome. J. Perinatol..

[12] Brar P.C., Heksch R., Cossen K., De Leon D.D., Kamboj M.K., Marks S.D., Marshall B.A., Miller R., Page L., Stanley T. (2020). Management and appropriate use of diazoxide in infants and children with hyperinsulinism. J. Clin. Endocrinol. Metab..

[13] Gray K.D., Dudash K., Escobar C., Freel C., Harrison T., McMillan C., Puia-Dumitrescu M., Cotten C.M., Benjamin R., Clark R.H. (2018). Prevalence and safety of diazoxide in the neonatal intensive care unit. J. Perinatol..

[14] Thornton P., Truong L., Reynolds C., Hamby T., Nedrelow J. (2019). Rate of serious adverse events associated with diazoxide treatment of patients with hyperinsulinism. Horm. Res. Paediatr..

[15] Collins L.C., Daniel K.B., Tolia V.N., Parikh P., Gray K.D., Greenberg R.G. (2026). Prevalence and safety of diazoxide in the neonatal intensive care unit. J. Perinatol..

[16] Herrera A., Vajravelu M.E., Givler S., Mitteer L., Avitabile C.M., Lord K., De León D.D. (2018). Prevalence of adverse events in children with congenital hyperinsulinism treated with diazoxide. J. Clin. Endocrinol. Metab..

[17] Newman-Lindsay S., Lakshminrusimha S., Sankaran D. (2023). Diazoxide for neonatal hyperinsulinemic hypoglycemia and pulmonary hypertension. Children.

[18] Duggal M., Moore S.S., Simoneau J., Girard G., Gernet I.B., Oettingen J.E.V., Sant’Anna G., Altit G. (2024). Pulmonary hypertension and necrotizing enterocolitis in neonates treated with diazoxide. Am. J. Perinatol..

[19] von Elm E., Altman D.G., Egger M., Pocock S.J., Gotzsche P.C., Vandenbroucke J.P., Initiative S. (2007). The Strengthening the Reporting of Observational Studies in Epidemiology (STROBE) statement. Lancet.

[20] Schachter Davidov A., Elkon-Tamir E., Haham A., Shefer G., Weintrob N., Oren A., Lebenthal Y., Mandel D., Eyal O. (2020). Higher C-peptide levels and glucose requirements may identify neonates with transient hyperinsulinism hypoglycemia who will benefit from diazoxide treatment. Eur. J. Pediatr..

[21] Kelly A., Tang R., Becker S., Stanley C.A. (2008). Poor specificity of low growth hormone and cortisol levels during fasting hypoglycemia for the diagnoses of growth hormone deficiency and adrenal insufficiency. Pediatrics.

[22] Laing D., Walsh E.P.G., Alsweiler J.M., Hanning S.M., Meyer M.P., Ardern J., Cutfield W.S., Rogers J., Gamble G.D., Chase J.G. (2024). Diazoxide for severe or recurrent neonatal hypoglycemia: A randomized clinical trial. JAMA Netw. Open.

[23] Balachandran B., Mukhopadhyay K., Sachdeva N., Walia R., Attri S.V. (2018). Randomised controlled trial of diazoxide for small for gestational age neonates with hyperinsulinaemic hypoglycaemia provided early hypoglycaemic control without adverse effects. Acta Paediatr..

[24] Malhotra N., Yau D., Cunjamalay A., Gunasekara B.S.A., Gilbert C., Morgan K., Dattani M., Dastamani A. (2024). Low-dose diazoxide is safe and effective in infants with transient hyperinsulinism. Clin. Endocrinol..

[25] Chen X., Feng L., Yao H., Yang L., Qin Y. (2021). Efficacy and safety of diazoxide for treating hyperinsulinemic hypoglycemia: A systematic review and meta-analysis. PLoS ONE.

[26] Keyes M.L., Healy H., Sparger K.A., Orth L.E., Geha M., Roumiantsev S., Matute J.D. (2021). Necrotizing enterocolitis in neonates with hyperinsulinemic hypoglycemia treated with diazoxide. Pediatrics.

[27] Prado L.A., Castro M., Weisz D.E., Jain A., Belik J. (2021). Necrotising enterocolitis in newborns receiving diazoxide. Arch. Dis. Child. Fetal Neonatal Ed..

[28] Solís-García G., Yeung T., Jasani B. (2023). Does the use of diazoxide for hyperinsulinaemic hypoglycaemia increase the risk of necrotising enterocolitis in neonates?. Arch. Dis. Child..

[29] Austin P.C., Stuart E.A. (2015). Moving towards best practice when using inverse probability of treatment weighting using the propensity score to estimate causal treatment effects in observational studies. Stat. Med..

