# Supplementary material for: Clinical and Hormonal Characteristics and Short-Term Outcomes of Diazoxide Use in Hypoglycemic Neonates: A Retrospective Cohort Study
Source: Children (Basel). 2026 Aug 28;13(9):1157. doi: 10.3390/children13091157 (PMC13605577; doi:10.3390/children13091157)
Supplement: Supplementary file 1 [file children-13-01157-s001.zip › children-4489568-supplementary.pdf]

Supplementary Material

Clinical and Hormonal Characteristics and Short-Term Outcomes of Diazoxide Use in Hypoglycemic Neonates: A Retrospective Cohort Study

Supplementary Table S1. Propensity-score sensitivity diagnostics.

Propensity covariate	Unweighted absolute SMD	Trimmed IPTW absolute SMD
Gestational age	0.340	0.130
Birth weight	0.294	0.094
Male sex	0.405	0.220
SGA	0.028	0.045
Maternal diabetes	0.491	0.072
Preeclampsia	0.480	0.049
Antenatal steroids	0.566	0.079
Severe hypoglycemia	0.257	0.034

Propensity score model: gestational age, birth weight, sex, SGA, maternal diabetes, preeclampsia, antenatal steroid exposure, and severe hypoglycemia. Propensity scores ranged from 0.052 to 0.896. Stabilized untrimmed weights ranged from 0.324 to 3.131; the 1st and 99th percentile trimming limits were 0.366 and 2.450. Effective sample size after trimming was 106.3 overall, 28.8 in the diazoxide group, and 77.4 in the untreated group. Absolute SMD <0.10 was prespecified as acceptable balance; gestational age and male sex remained above this threshold.

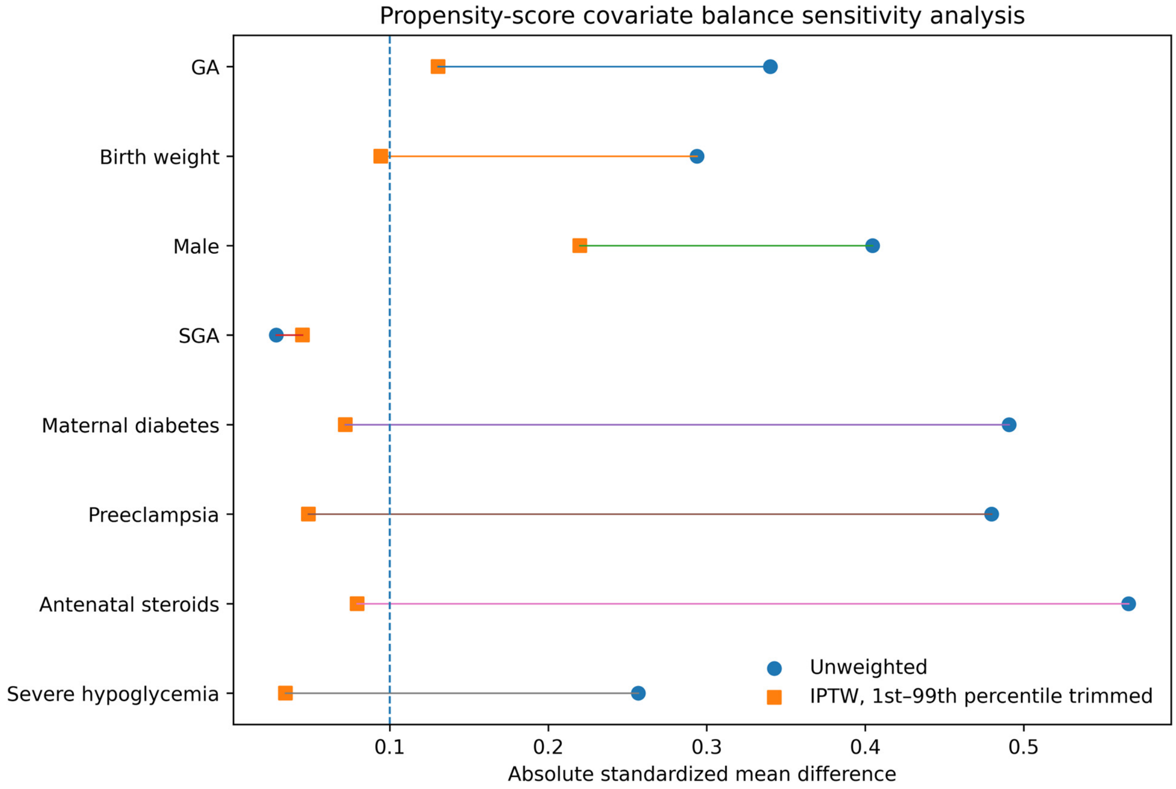

Supplementary Figure S1. Propensity-score covariate balance before and after 1st-99th percentile trimmed stabilized IPTW. The dashed vertical line indicates an absolute SMD of 0.10.

Interpretation: Trimmed IPTW improved balance for most baseline variables but did not achieve the prespecified SMD <0.10 criterion for gestational age or male sex. Because the untreated group also lacked a treatment-index time equivalent to diazoxide initiation, post-treatment weighted clinical-outcome comparisons were not retained as causal estimates in the main analysis.
